# The Prognostic and Therapeutic Value of PD-L1 in Glioma

**DOI:** 10.3389/fphar.2018.01503

**Published:** 2019-01-09

**Authors:** Ruo Qiao Chen, Feng Liu, Xin Yao Qiu, Xiao Qian Chen

**Affiliations:** ^1^School of Basic Medicine, Tongji Medical College, Huazhong University of Science and Technology, Wuhan, China; ^2^Department of Pathophysiology, School of Basic Medicine, Tongji Medical College, Huazhong University of Science and Technology, Wuhan, China

**Keywords:** glioma, PD-L1, immune response, tumor infiltrating lymphocytes, Ras

## Abstract

Glioma is the most common type of primary brain tumors. After standard treatment regimen (surgical section, radiotherapy and chemotherapy), the average survival time remains merely around 14 months for glioblastoma (grade IV glioma). Recent immune therapy targeting to the immune inhibitory checkpoint axis, i.e., programmed cell death protein 1 (PD-1) and its ligand PD-L1 (i.e., CD274 or B7-H1), has achieved breakthrough in many cancers but still not in glioma. PD-L1 is considered a major prognostic biomarker for immune therapy in many cancers, with anti-PD-1 or anti-PD-L1 antibodies being used. However, the expression and subcellular distribution of PD-L1 in glioma cells exhibits great variance in different studies, severely impairing PD-L1's value as therapeutic and prognostic biomarker in glioma. The role of PD-L1 in modulating immune therapy is complicated. In addition, endogenous PD-L1 plays tumorigenic roles in glioma development. In this review, we summarize PD-L1 mRNA expression and protein levels detected by using different methods and antibodies in human glioma tissues in all literatures, and we evaluate the prognostic value of PD-L1 in glioma. We also summarize the relationships between PD-L1 and immune cell infiltration in glioma. The mechanisms regulating PD-L1 expression and the oncogenic roles of endogenous PD-L1 are discussed. Further, the therapeutic results of using anti-PD-1/PD-L1 antibodies or PD-L1 knockdown are summarized and evaluated. In summary, current results support that PD-L1 is not only a prognostic biomarker of immune therapy, but also a potential therapeutic target for glioma.

## Introduction

Glioma, predominantly derived from glial cells, is the most common type of primary tumors in the human brain. Pathologically, glioma is categorized into grade I-IV according to World Health Organization (WHO) criteria, in which grade I-II are considered low-grade glioma (LGG) while grade III-IV are high-grade glioma (HGG). WHO grade IV glioma, also called glioblastoma or glioblastoma multiforme (GBM), is characterized by its tissue ischemic necrosis, strong invasiveness and microvascular proliferation. After conventional surgery, radiation therapy and chemotherapy, GBM patients have a 5-year survival rate of around 9.8% and a median survival time of 14 months (Stupp et al., 2005, 2009; Tran and Rosenthal, 2010). Currently, there is still no effective clinical therapy for primary or recurrent GBM. Recently, immune therapies targeting to the inhibitory immune checkpoint axis signaling, mainly PD-1/PD-L1 pairs, are revolutionary. Blocking PD-1/PD-L1 pathway by antibodies achieved clinical cure in advanced melanoma including its brain metastasis (Wolchok et al., 2017; Long et al., 2018), opening a new era of cancer therapies. Pembrolizumab and Nivolumb, the most commonly used monoclonal PD-1 antibodies approved by USFDA, have been widely used for melanoma as well as non-small cell lung cancer (Robert et al., 2015; Reck et al., 2016). Currently, many other cancers such as renal carcinoma, colorectal cancer, lymphoma, head and neck carcinoma, bladder cancer, hepatocellular carcinoma and metastatic colorectal cancer are also approved for PD-1/PD-L1-targeting therapy (Ansell et al., 2015; Le et al., 2015; Ferris et al., 2016; Bellmunt et al., 2017; Motzer et al., 2018; Overman et al., 2018). The predictive markers in PD-1/PD-L1 antibody therapy are mainly the number of cytotoxic T-lymphocytes inside tumor tissues and the expression level of PD-L1 in cancer cells (Chen et al., 2016). Clinical trials of PD-1/PD-L1 antibody immunotherapy for glioma are relatively delayed, largely remaining on phase II (e.g., NCT01952769, Pidilizumab) and phase III (e.g., NCT02017717, Nivolumab) (Filley et al., 2017; Maxwell et al., 2017). Till now, there is still no reliable predicative marker for targeting inhibitory immune checkpoint in glioma. Meanwhile, unlike other solid tumors, the relationship between PD-L1 and T-lymphocyte infiltration in glioma, as well as therapeutic effects of PD-1/PD-L1 antibodies remains largely elusive, which probably reflect the specificities of cellular and structural microenvironment in the brain (Huang et al., 2017). Here, we summarize major recent results: (1) PD-L1 expression/subcellular distribution in glioma tissues; (2) The correlation between PD-L1 level and survival time of glioma patients; (3) The relationship between PD-L1 and immune cell infiltration; (4) The mechanism controlling PD-L1 expression and its intracellular oncogenic role; (5) The therapeutic effects of PD-1 antibodies in patients and PD-L1 antibodies in animals.

## The Expression and Subcellular Distribution of PD-L1 in Human Glioma Tissues

PD-1 and PD-L1 are two major negative regulatory molecules at the immune checkpoint axis. PD-1 (i.e., CD279), a cell surface receptor belonging to the extended CD28/cytotoxic T-lymphocyte-associated protein 4 (CTLA-4) family of T cell regulators (Shinohara et al., 1994), is predominantly expressed on activated T cells, B cells, and macrophages (Agata et al., 1996). PD-L1 and PD-L2, both PD-1 ligands, belongs to the B7 family. PD-L1 protein is widely expressed in almost all tumor cells as well as many normal cells, while PD-L2 is mainly expressed in dendritic cells and a few tumor cell lines. Crucially, PD-L1 can be upregulated in cancer cells upon interferon-γ (IFN-γ) stimulation and some activated immune cells (e.g., macrophages, dendritic cells) (Dong et al., 1999), indicating the major regulator role of PD-L1 in cancer tissues. PD-L1 binds not only to PD-1 but also other costimulatory molecules such as CD28, CD80, and CTLA-4, whereas PD-L2 binds mainly to PD-1 (Said et al., 2010), suggesting that PD-L1 has wider and more complicated mechinisms for regulating immune responses. The binding of PD-L1 to PD-1 delivers strong inhibitory signals to suppress the proliferation, activation and infiltration of cytotoxic T-lymphocytes (CTL) (Dong et al., 2002), which was proved to be the major negative regulation of CTL in cancer microenvironment (Dong et al., 2002; Alsaab et al., 2017; Wang et al., 2018). Since PD-L1 level in cancer cells is considered to be a major predictive marker of PD-1/PD-L1 antibody response (Sanmamed and Chen, 2018), it is of primary importance to analyze the expression and subcellular distribution of PD-L1 in glioma tissues.

In previous studies, flow cytometry (FCM) detected PD-L1 expression in 12 glioma cell lines (Wintterle et al., 2003), most primary cultures of glioma cells (6/8) (Wilmotte et al., 2005) and glioma cells from a great number of human glioma specimens (Berghoff et al., 2015), demonstrating the presence of PD-L1 on cell surface of most glioma cells. In addition, many results of immunohistochemistry (IHC) show much higher PD-L1 protein expression in human glioma tissues than that in their surrounding or distant normal tissues (Berghoff et al., 2015; Wang and Wang, 2017). However, PD-L1 levels in human glioma tissues detected by different laboratories vary greatly (Table 1). It is worth noting that 11 different PD-L1 antibodies have been utilized in IHC, and each PD-L1 antibody showed distinct PD-L1 expression and subcellular distribution pattern in glioma cells, indicating the intriguing role of PD-L1 in glioma.

**Table 1:** The expression of PD-L1 in human glioma tissues.

**Materials**	**PD-L1 antibody**	**Criteria for IHC positive**	**Expression rate of PD-L1**	**Other results**	**Reference**
TMA:1035 GBM specimens (grade IV tumors with gliosarcoma were not included)	SP142	<5% or <2+ or ≥2+ and ≥5%	PD-L1 positive in 19% of all specimens		Xiu et al., 2016
233 GBM specimens; WHO I/II, 15.6%; WHO III 13.6%; gliosarcomas 3.7%	SP142	PD-L1 expression detected on cell membranes	PD-L1 positive in 6.1% (21/345) of all glioma specimens, and in 35.0% (57/163) of all GBM specimens	High expression of PD-L1 in WHO IV GBM specimens, while low expression in IDH-mutant glioma specimens	Garber et al., 2016
327 glioma specimens (grade I-IV); 198 GBM specimens	SP142	PD-L1 expression detected on cell membranes	PD-L1 positive in 8.1% (24/295) of all glioma specimens, and in 10.1%(19/189) of all GBM specimens	Low expression of PD-L1 in IDH-mutant glioma specimens	Hodges et al., 2017
117 newly diagnose glioblastoma specimens, 18 recurrent glioblastoma specimens	Clone 5H1	Membranous PD-L1 expression: PD-L1 detected on >5% of the tumor cell membranes;	PD-L1 positive in 37.6% (44/117) of newly diagnosed glioblastoma specimens and 16.7%(3/18) of recurrent glioblastoma specimens;		Berghoff et al., 2015
		Diffuse/fibrillary PD-L1 expression: PD-L1 detected in non-necrotic areas	PD-L1 expression in 84.6% (99/117) of newly diagnosed glioblastoma specimens and 72.2% (13/18) of recurrent glioblastoma specimens.		
43 WHO II/III (39 IDH-mutant, 4 IDH-wild type) glioma specimens; 14 GBM with IDH-mutant specimen; 117 GBM with IDH-wild type specimens	Clone 5H1	Membranous PD-L1 expression: PD-L1 detected on >1% of the tumor cell membranes;	Positive expression in 56.2% (68/121) of IDH-wild type glioma and 5.7% (3/53) of IDH-mutant glioma;	A significant negative correlation between PD-L1 expression and IDH-mutant status (P < 0.001)	Berghoff et al., 2017
		Diffuse/fibrillary PD-L1 expression: PD-L1 detected in >25% of all non-necrotic areas	Positive expression in 84.3% (102/121) of IDH-wild type glioma and 37.3%(20/53) of IDH-mutant glioma.		
9 GBM specimens; 1 mixed glioma (WHO III) specimen	Clone 5H1	PD-L1 expression was divided into 4 levels: <25%; 25–50%; 50–75% and >75%	PD-L1 expression in all 10 specimens (with a positive rate of 50~90%)		Wintterle et al., 2003
TMA: 99 GBM specimens	EPR1161	Compact brown particles shown on cell membranes	60.6% of the specimens with >1% PD-L1 expression; 38.3% of the specimens with >5% PD-L1 expression		Nduom et al., 2016
TMA: 229 glioma specimens (WHO I~IV)	Rabbit polyclonal antibody anti-PD-L1	>5% of tumor cell with membrane or cytoplasm PD-L1 expression	49.2, 53.7, and 68.6% of grade II, III and IV gliomas with positive PD-L1 expression respectively	No significant correlation between PD-L1 expression and WHO levels (*P* = 0.327)	Zeng et al., 2016
54 brain tumor specimens, 1 epilepsy specimen	clone MIH1 eBioscience	Positive signals detected in glioma cells	Positive expression of PD-L1 in 85.2%(46/54) of all brain tumor specimens and 19/19 GBM specimens	The positive expression rate of PD-L1 in GBM specimens higher than that in other brain tumor specimens	Wilmotte et al., 2005
64 glioma specimens (grade I-IV)	ab58810, Abcam	Positive signals detected in>5% of all tumor cells (membrane or cytoplasm)	PD-L1 positive in 78.12% (50/64) of all specimens, 60.87% (14/23) of the LGG specimens and 87.80%(36/41) of the HGG specimens	A significant positive correlation between PD-L1 expression and WHO levels (*P* = 0.013)	Xue et al., 2017a
Initial and secondary resected tumor specimens from 16 GBM patients (excision time within 2 years after the first operation)	28–8, Abcam	PD-L1 in tumor cells was graded as “–” (absence of staining), “+” (up to 25% of cells stained), “++” (25–50% of cells stained) or “+++” (more than 50% of cells stained)	Initial resected tumor specimens median:++; secondary resected tumor specimens median:+++	No difference between the expression of PD-L1 in initial and secondary resected tumor (*P* = 0.187)	Miyazaki et al., 2017
TMA: 54 GBM specimens	Cell Marque, Rocklin, CA, USA	PD-L1 expression detected in >5% of all tumor cell membranes	PD-L1positive in 31.5% of all GBM specimens		Han et al., 2017
Initial and secondary resected tumor specimens from 64 GBM	E1LRN, Cell Signaling	Diffuse/fibrillary PD-L1 expression in 75% of all specimens; Cytoplasmic PD-L1 expression in 20% of all specimens; membranous PD-L1expression in 5% of all specimens	PD-L1 expression level in secondary resected GBM specimens reduced 66.71% compared to that in initial resected GBM specimens (*p* = 0.0045)		Heynckes et al., 2017
62 malignant brainstem glioma specimens	ab205921, Abcam, Cambridge, UK	Unknown	PD-L1 expression in 59.7% (37/62) of all specimens		Zhang et al., 2017
TMA: 115 GBM specimens	E1L3N, Cell Signaling	Positive signals detected in >5% of tumor cells (either membrane or cytoplasm)	PD-L1 positive in 32.2% (37/115) of all GBM specimens	PD-L1 expression was significantly associated with IDH-mutant status (*P* = 0.008)	Lee et al., 2018

*TMA,tissue microarray; GBM, glioblastoma multiforme; IDH-mutant, isocitrate dehydrogenase-mutant*.

First of all, the most widely used PD-L1 antibody Clone 5H1 can detect both diffuse/fibrillary PD-L1 intracellularly and on cell surface (Wintterle et al., 2003; Berghoff et al., 2015; Bellmunt et al., 2017). In two experiments involving 9 GBM and 1 grade III mixed glioma specimens, PD-L1 protein was detected in all glioma samples and areas with PD-L1^+^-cells accounted for 50–90% of the specimens (Wintterle et al., 2003). In another study containing 174 human glioma specimens, Clone 5H1 showed that 70.1% of all specimens was diffuse/fibrillary PD-L1-positive (>25% of the tumor cells was PD-L1^+^), and 40.8% of all specimens was PD-L1^+^ on cell membranes (Berghoff et al., 2017). Whereas, in a study containing 135 glioma specimens, only 34.8% of all glioma specimens were PD-L1^+^ (Berghoff et al., 2015). Moreover, most glioma specimens (82.9%) exhibited a diffuse/fibrillary distribution pattern of PD-L1, while membranous distribution of PD-L1 in tumor cells covered >5% merely (Berghoff et al., 2015). In short, the employment of Clone 5H1 receives high positive rates of PD-L1 in human glioma tissues (>30%).

When using PD-L1 antibody SP142, the percentage of PD-L1^+^ samples decreases greatly. In all three studies involving large numbers of specimens, the detected rates of PD-L1 were <20%: 1. PD-L1 was positive in 19% of all 1035 GBM specimens (≥2+ and ≥5%) (Xiu et al., 2016); 2. PD-L1 was positive in only 6.1% of all 347 glioma specimens (staining of PD-L1 on tumor cell membranes >1+) (Garber et al., 2016); 3. PD-L1 was positive in only 8.1% of all 327 glioma specimens (staining of PD-L1 on tumor cell membranes >1+) (Hodges et al., 2017).

Furthermore, Nduom et al. (2016) compared several PD-L1 antibodies including Abcam ab58810, Abcam clone EPR1161, Clone 7G11, and Clone 5H1 antibodies. They found that PD-L1 antibody EPR1161 showed relatively satisfying results of membranous PD-L1. The specificity of EPR1161 antibody to PD-L1 was confirmed by several positive controls (e.g., human placenta, tonsil tissues and PD-L1-overexpressed HEK 293 cells). EPR1161 antibody detection was used to analyze PD-L1 expression on a tissue microarray containing 99 GBM specimens. Only tumor cells showed positive PD-L1 signals on cell membranes: 60.6% of all specimens contained >1% PD-L1^+^-glioma cells, 38.3% contained >5% PD-L1^+^-glioma cells, 17% contained >25% PD-L1^+^-glioma cells and 5.32% contained >50% PD-L1^+^-glioma cells. These results indicate that a more comprehensive evaluation of PD-L1 expression in glioma is desirable.

Importantly, several recent studies reported that expression levels of PD-L1 are positively correlated with glioma grades (Garber et al., 2016; Wang et al., 2016; Xue et al., 2017a; Zhang et al., 2017). Xue et al. (2017a) reported that the PD-L1 expression levels positively correlated with the grades of gliomas. Wang et al. (2016) and Zhang et al. (2017) reported that PD-L1 expression was much higher in GBM compared to grade II and III gliomas. Garber et al. (2016) found that the high expression level of PD-L1 (IHC) was positively associated with only grade IV gliomas, though high PD-L1 expression could also be detected in other grades of gliomas.

Moreover, PD-L1 expression is associated with glioma genotypes. For instance, in different grades of gliomas, all PD-L1 expression in isocitrate dehydrogenase (IDH)-mutant gliomas was significantly lower than that in IDH-wild type (Wang et al., 2016). In fact, the methylation level of PD-L1 promoter in IDH-mutant glioma was higher than that in IDH-wild type glioma, and PD-L1 expression negatively correlated with PD-L1 promoter methylation level (Berghoff et al., 2017; Röver et al., 2018). Additionally, low PD-L1 levels (IHC) were observed in proneural and glioma CpG island methylator phenotype (G-CIMP) subtypes, while high PD-L1 expression was detected in mesenchymal subtype (Berghoff et al., 2015).

In brief, the expression patterns of PD-L1 in specimens are mainly influenced by the following factors: (1) The selected PD-L1 antibodies: if the antibody can detect both membranous and cytoplasmic PD-L1, the percentage of PD-L1-positive specimens rises remarkably [e.g., Clone 5H1 (Wintterle et al., 2003; Berghoff et al., 2015, 2017), Clone MIH1 eBioscience (Wilmotte et al., 2005), ab58810 Abcam (Xue et al., 2017a), E1LRN Cell Signaling (Heynckes et al., 2017), ab205921 Abcam (Zhang et al., 2017), and E1L3N Cell Signaling (Lee et al., 2018)]; if the antibody detects only membranous PD-L1, the percentage of PD-L1-positive specimens is low (e.g., SP142,EPR1161) (Garber et al., 2016; Nduom et al., 2016). (2) The PD-L1-positive criteria: if membranous PD-L1 is used as criteria, low PD-L1-positive percentage appears; while if cytoplasmic PD-L1 (either diffuse or fibrillary pattern) serves as criteria, much higher PD-L1-positive percentage can be obtained. (3) The constitution of illness cases: the expression of PD-L1 is considerably increased in GBM specimens, but decreased in recurrent glioma specimens (Berghoff et al., 2015) as well as IDH-mutant glioma specimens. (4) The stability of PD-L1 on glioma cell membranes: PD-L1 can be detected at various cellular components such as cell membrane, cytoplasm or vesicles. It is conceivable that the recycling pattern and turnover rate of PD-L1 in glioma cells can affect PD-L1 detection.

## The Prognostic Value of PD-L1 in Glioma Patients

Many studies investigated the correlation between PD-L1 expression levels and prognosis of glioma patients. In brief, more than half of literatures reported a negative correlation between PD-L1 and the prognosis of glioma patients. However, others reported that PD-L1 was not correlated with the prognosis of glioma (Table 2). Basing on The Cancer Genome Atlas (TCGA) and Chinese Glioma Genome Atlas (CGGA) database analysis and meta-analysis, in two studies involving 976 and 1,052 glioma patients respectively, high PD-L1 mRNA expression level was found to be associated with significantly shorter overall survival (OS) of glioma patients (Wang et al., 2016; Xue et al., 2017b). Various other studies basing on IHC results reported similar relationship (Liu et al., 2013; Nduom et al., 2016; Han et al., 2017; Lee et al., 2018; Pratt et al., 2018). Whereas in several other IHC analyses as well as a TCGA database analysis involving 444 GBM patients, no significant relationship was found between PD-L1 expression and glioma patients' OS (Berghoff et al., 2015; Zeng et al., 2016; Miyazaki et al., 2017). This is probably due to their relatively small scale of glioma samples.

**Table 2:** The relationship between PD-L1 expression and prognosis of glioma patients.

**Materials**	**PD-L1 antibody**	**Correlation with prognosis**	**Reference**
Data of 149 GBM patients from TCGA		TCGA database analysis: for PD-L1 and PD-1, high expression associated with significantly shorter survival (Kaplan-Meier, *P* = 0.023 and *P* = 0.028, respectively); high expression of both PD-L1and PD-1 negatively correlates with prognosis of patients (*P* = 0.0031)	Nduom et al., 2016
976 glioma specimens' data from CGGA and TCGA		CGGA database analysis: negative correlation between PD-L1 expression and the OS of glioma patients (P < 0.001) or GBM patients (*P* = 0.0253); TCGA database analysis: negative correlation between PD-L1 expression and the OS of glioma patients (P < 0.001) or GBM patients (*P* = 0.043)	Wang et al., 2016
TMA: 229 glioma specimens (grade I-IV)	Rabbit polyclonal antibody	No significant correlation between PD-L1 expression and patients' OS; univariate analysis: negative correlation between PD-L1 expression and prognosis of patients whose OS > 12 months (*P* = 0.018); multivariate analysis: no significant relation between PD-L1 expression and poor OS, but a strong tendency toward significance.	Zeng et al., 2016
117 newly diagnosed glioma specimens, 18 recurrent glioma specimens	Clone 5H1	No significant correlation between PD-L1 expression and patients' OS (P = 0.724)	Berghoff et al., 2015
Initial and secondary resected glioma specimens from 16 GBM patients (time of resection within 2 years after the first operation)	28-8, Abcam	No significant correlation between PD-L1/PD-1 expression and patients' PFS or OS in initial resected specimens; PD-1 high expression significantly associated with long progression-free survival (*P* = 0.029) and short survival after recurrence (*P* = 0.030) in secondary resected specimens	Miyazaki et al., 2017
TMA: 54 GBM specimens	Cell Marque, Rocklin, CA, USA	Negative correlation between PD-L1 expression and patients' OS (multivariate analysis: *P* = 0.007; univariate analysis: *P* = 0.024; Kaplan-Meier survival analysis: *P* = 0.017); no significant correlation between PD-L1 expression and patients' PFS (*P* = 0.14)	Han et al., 2017
TMA: 115 GBM specimens	E1L3N, Cell signaling	Kaplan–Meier analysis: PD-L1 expression in tumor cells significantly associated with poor overall survival (OS) (P = 0.017)	Lee et al., 2018
TMA: 99 GBM specimens	EPR1161	Significant negative correlation between PD-L1 expression and patients' OS (*P* = 0.086)	Nduom et al., 2016
Gene methylation data of 419 LGG patients from TCGA		Positive correlation between PD-1 methylation and patients' OS (*P* = 0.001); no significant correlation between PD-L1 methylation and the prognosis of patients	Röver et al., 2018
Data of 1,052 glioma patients from 4 previous studies		Meta analysis: pooled result: high PD-L1 expression associated with worse OS in glioma patients (*P* = 0.032); subgroup analysis: high PD-L1 expression in glioblastoma (GBM) associated with worse OS (*P* = 0.030); index subgroup analysis: neither PD-L1 protein (*P* = 0.068) nor gene (*P* = 0.322) significantly associated with OS	Xue et al., 2017b
Specimens from 17 GBM patients	MIH5	PD-L1 expression in tumors negatively associated with GBM patient survival (*P* = 0.001)	Liu et al., 2013
TMA: 183 tumor patient tissues (102 IDH-wild type diffuse gliomas);		NIH cohort glioblastoma and NIH cohort recurrent glioblastoma (IDH-wildtype): PD-L1 negatively associated with patients' OS (*P* < 0.001 and *P* = 0.015, respectively);	Pratt et al., 2018
Data of 444 GBM and 12 recurrent, non–G-CIMP (IDH-wild type) samples from TCGA		TCGA database analysis: in all glioblastoma, no significant correlation between PD-L1 expression and patients' OS (*P* = 0.135); in recurrent, non–G-CIMP (IDH-wild type), PD-L1 expression negatively associated with patients' OS (*P* = 0.023)	
Data of 47 GBM patient samples from a previous clinical study		GBM tumor cells expressing PD-L1 nor not does not affect the OS and PFS of ADCTA group or reference group patients (ADCTA group OS *P* = 0.086 and PFS *P* = 0.239; reference group OS *P* = 0.376 and PFS *P* = 0.421)	Jan et al., 2018

*TABT, tumor-adjacent brain tissue; LGG, low-grade glioma*.

In addition to case number, PD-L1^+^-cellular components in glioma microenvironment may also be an important factor affecting the prognostic value of PD-L1. Several studies reported that PD-L1 in glioma microenvironment is contributed mainly by tumor-infiltrating myeloid cells (TIM, including macrophages and T-regulatory cells) rather than tumor cells themselves (Mirghorbani et al., 2013; Antonios et al., 2017). The increase of PD-L1^+^ TIM surrounding glioma cells is associated with strong immune inhibition (Liu et al., 2008; Mirghorbani et al., 2013; Hosseini et al., 2015). Jan et al. (2018) found that lower PD-L1 level in glioma cells is associated with neither abating immune inhibition nor better prognosis of glioma, which is probably linked with elevated PD-L1^+^ TIM in glioma microenvironment.

Constitution of illness cases and limited human glioma tissue may also influence the evaluation of PD-L1 as a prognostic marker. For instance, the following factors can be taken into account: (1) The glioma type. Pratt et al. (2018) reported that there was no significant correlation between PD-L1 expression and OS (*P* = 0.135) of all glioma patients, while among recurrent, non–G-CIMP (IDH-wild type) patients, the PD-L1 expression was negatively associated with the OS (*P* = 0.023). This evidence suggests that PD-L1 expression may have prognostic values in specific subtypes of gliomas. Future studies may focus on analyzing the relationships between PD-1 and other molecular markers of glioma. (2) The way of resection. In a study involving initial and secondary resected glioma specimens from 16 GBM patients, PD-1 but not PD-L1 expression was positively associated with long progression-free survival (PFS) (*P* = 0.029) in initial resected specimens. After secondary surgery, PD-1 expression was negatively associated with survival time following recurrence (*P* = 0.030) (Miyazaki et al., 2017). (3) The time of patients' survival. In Zeng's study (Zeng et al., 2016) involving 229 glioma specimens of grade I-IV, no significant correlation between PD-L1 and OS was found in all gliomas, but further analysis revealed a negative correlation between PD-L1 and OS (*P* = 0.018) if OS > 12 months is set a criterion in grade IV glioma only. While for those whose OS are <12 months, PD-L1 expression was not significantly associated with patients' OS, suggesting that PD-L1 is more favorable for the prediction of long-time survival patients (Zeng et al., 2016).

Although less studied, PD-1 expression level was also reported to be inversely associated with prognosis of GBM patients (Wang et al., 2016). In addition, PD-1 but not PD-L1 methylation was found to be positively associated with the OS of LGG patients (Röver et al., 2018). What is more, Bloch et al. (2017) reported that lower PD-L1 expression on peripheral myeloid cells is a primary independent predictor of survival in GBM patients (n = 46) receiving autologous heat-shock protein vaccine (Prophage) after surgical resection followed by standard radiation and chemotherapy. The evaluation of PD-L1 expression on peripheral blood cells has great practical applications in predicting patients' survival time and their responses to PD-1/PD-1 antibody therapy. Clearly, the predicting value of PD-L1 or PD-1 in glioma will be increased with accumulating data. In addition, the search of other biomarkers is also important (Xue et al., 2017c).

## PD-L1 Modulating Immune Cell Infiltration in Glioma Microenvironment

As is well-known, the engagement of PD-1 and PD-L1 promotes apoptosis of PD-1^+^ CTL. In various types of cancers including glioma, PD-L1 plays a major inhibitory role in modulating infiltration of immune cells, such as CTL, tumor infiltrating lymphocytes (TIL) and regulatory T-lymphocytes (Treg) (Taube et al., 2012; Dong et al., 2017). Thus it is the rationale to hypothesize that inhibiting PD-1/PD-L1 signaling promotes the antitumoral activity of TIL. Indeed, this hypothesis has been widely demonstrated in various cancer models including glioma (Taube et al., 2012; Antonios et al., 2016; Dong et al., 2017) and proved to be clinically effective in several cancers such as melanoma and non-small cell lung cancer. In fact, the relationships between PD-L1^+^ cancer cells and PD-1^+^ immune cells (mainly TIL) in tumor environment are complicated. For example, TIL secrete large amounts of cytokines (e.g., IFN-γ) to strongly upregulate PD-L1 expression in melanoma cells, which in turn triggers TIL themselves' inhibition (Taube et al., 2012). In most cancers, the upregulation of PD-L1 in tumor cells appears to be associated with increased TIL (Antonios et al., 2016).

PD-L1 plays opposite regulatory roles in functions of CTL and Treg in various cancers. Early studies considered that the interaction of PD-L1/tumor cells and PD-1/CTL may form a temporal “PD-L1/PD-1 shield” to prevent CTL-mediated tumor cell lysis (Hirano et al., 2005). In this model, it is supposed that CTL lose their ability to control tumor growth but remain intact. Later, it was discovered that the PD-1/PD-L1 interaction increases indoleamine 2,3-dioxygenase (IDO) in melanoma microenvironment, which exhausts T cells of essential tryptophan and suppresses their metabolites, thus leading to CTL inhibition and Treg elevation (Dong et al., 2017). Moreover, PD-L1 inhibits chemokine production (e.g., CCL2-5 and CXCL9-10) that is crucial for recruitment of CTL, preventing the influx of CTL into tumor microenvironment (Gajewski et al., 2013). The roles of PD-1/PD-L1 pathway in inducing CTL exhaustion and Treg augment in tumor microenvironment have been verified in various cancer models (Dong et al., 2017; Jan et al., 2018).

PD-L1 in gliomas shows similar effects on CTL and Treg as in other cancers. Early studies demonstrated that PD-L1 expressed by glioma cells inhibits T-lymphocyte functions and induces apoptosis of tumor-specific T-lymphocytes mainly via decreasing cytokines production (e.g., IFN-γ, IL-2 and IL-10) (Wintterle et al., 2003; Ahn et al., 2013). However, more recent studies revealed that PD-L1/PD-1 pathway inhibits CTL functions primarily via TIMs in glioma microenvironment (Antonios et al., 2017). The upregulation of PD-L1 in circulating monocytes and tumor-infiltrative macrophages in gliomas is associated with its cytotoxicity to T cells (Bloch et al., 2013). What is more, PD-L1 induces and maintains Treg in glioma. The elevation of PD-L1^+^ peripheral blood cells was associated with an increased Treg fraction in GBM patients (DiDomenico et al., 2018). Moreover, PD-L1 co-stimulation resulted in greater Treg expansion and improved preservation of the Treg phenotype, indicating that PD-L1 may expand and maintain immunosuppressive Treg. The increase of Treg is associated with decreased survival in glioma patients (DiDomenico et al., 2018). Until now, the exact relationships among PD-L1 and infiltrating immune cells in gliomas are far from clear. A major difficulty may be the very limited brain tissues obtained from glioma patients. Accurate information requires data accumulation from large quantity of human glioma samples.

## The Regulatory Mechanisms of PD-L1 Expression and Secretion

In gliomas, the tumor suppressor phosphate and tension homology deleted on chromsome 10 (PTEN), a major negative regulator of Akt activation, plays a vital role in regulating PD-L1 protein expression. PTEN mutation or homozygous deletion (in 36% of glioma) is highly associated with PD-L1 expression in gliomas. Activation of PI3K-Akt-mTOR-S6K1 cascade facilitates PD-L1 transcript into polysome so that PD-L1 translation increases (Parsa et al., 2007). Co-activation of Ras and Akt in glioma cells further elevated PD-L1 translation by recruiting polysome. PTEN loss in glioma causes Akt activation, thus induces PD-L1 protein via its translational regulatory mechanisms. On the contrary, the restoration of PTEN function reduces PD-L1 expression in gliomas. In addition to PTEN mutation, cytokines such as interferons may also induce PD-L1 via PI3K-Akt-mTOR-S6K1-mediated translational regulation (D'Arrigo et al., 2017).

Recently, microRNA-34a (miR-34a) and the PD-L1 co-chaperone FK506 binding protein 5 (spliced FKBP51) are also reported to be associated with PD-L1 expression in gliomas. MiR-34a functions as a tumor suppressor via modulating epidermal growth factor receptor (EGFR) or PD-L1 translation directly in glioma. miR-34a overexpression attenuated PD-L1-induced chemoresistance in glioma cells, supporting that miR-34a is a negative regulator of PD-L1 signaling (Wang and Wang, 2017). FKBP51s works as a co-chaperone with isomerase activity. FKBP51s abundantly exists in glioma and increases membranous PD-L1 by catalyzing the protein folding required for subsequent glycosylation. FKBP51s silencing significantly decreases PD-L1 on U251 cell surface. Moreover, FKBP51s is required for PD-L1 maturation in endoplasmic reticulum (ER), supporting that the FKBP51s functions as an important PD-L1 molecular chaperone (D'Arrigo et al., 2017).

IFN-γ is likely to be the strongest PD-L1 inducer in various cancers. It is reported that PD-L1 upregulation in melanoma tumor microenvironment depends on CTL-secreted IFN-γ rather than cancer cells themselves (Gajewski et al., 2013; Spranger et al., 2013; Dong et al., 2017). Some immunogenic tumors (e.g., metastatic colorectal cancer with high-level microsatellite instability, MSI-H-CRC) attract TIL, which produce IFN-γ and upregulate PD-L1 in tumor epithelial cells (Gatalica et al., 2014). In this way TIL can actually trigger their own inhibition by secreting cytokines that drive tumor PD-L1 expression (Taube et al., 2012). In gliomas, both monocytic and granulocytic myeloid-derived suppressor cells may contribute largely to PD-L1 upregulation inside the tumors, as infiltrated CTL were few (Dubinski et al., 2016).

IFN-γ relies on the activation of cyclin-dependent kinase 5 (Cdk5) to induce PD-L1 expression (Dorand et al., 2016). Loss of Cdk5 activity in many cancers results in persistent overexpression of the interferon regulatory factors IRF2 and IRF2BP2, two PD-L1 transcriptional repressors that negatively regulate PD-L1 expression in tumor cells. The persistent interferon regulatory factor 2 (IRF2) and IRF2-binding protein 2 (IRF2BP2) overexpression lasts for up to 48 hours after IFN-γ exposure in Cdk5-deficient medulloblastoma cells. Furthermore, disrupting Cdk5 in rhabdomyosarcoma also led to IFN-γ-induced PD-L1 ineffectiveness, supporting a general role of Cdk5 in PD-L1 regulation in cancer cells. Thus, Cdk5 is a key enzyme linked to IFN-γ and PD-L1 upregulation (Dorand et al., 2016).

IL-10 may serve as a vital cytokine upregulating PD-L1 expression in circulating monocytes and tumor-infiltrative macrophages in an autocrine/paracrine pattern in gliomas. Previous studies reported that glioma cells could stimulate monocytes and glioma-associated macrophages to produce IL-10, which in turn significantly increases PD-L1 expression in monocytes and tumor-infiltrative macrophages (Bloch et al., 2013).

**Figure 1:**
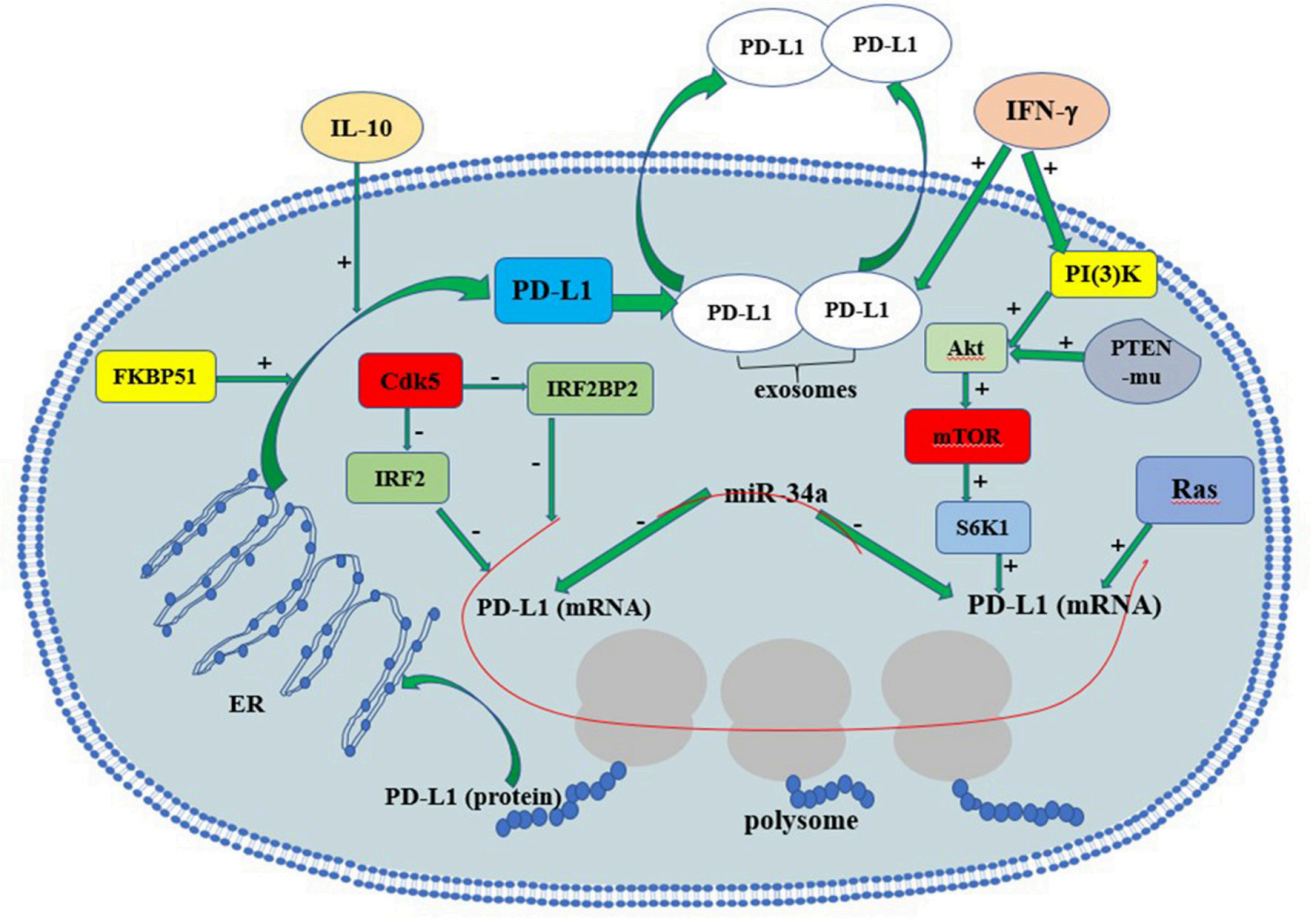
The regulation of PD-L1 expression in glioma cells. Red line indicates RNAs. PTEN-mu, PTEN-mutant; ER, endoplasmic reticulum.

Most recently, a novel mechanism of PD-L1-induced immune suppression is discovered, i.e., the interaction between interferon-γ-induced exosomal PD-L1 and PD-1 on peripheral T-cells (Chen et al., 2018). In metastatic melanomas cells, extracellular vesicles (mainly exosomes) with PD-L1 on exosome surface are secreted into tumor microenvironment and body circulation. IFN-γ exacerbates PD-L1's release in melanoma exosomes, suppressing nearby and remote CD8^+^-T cell functions and facilitating tumor growth. Such evidence suggests that circulating exosomal PD-L1 may be a more practical and dynamic monitoring-marker for predicting host responses to immune therapy. The regulatory mechanisms of PD-L1 expression and secretion are summarized in Figure 1. The precise understanding of detailed PD-L1 regulatory mechanisms may provide insight in developing novel immunotherapeutic strategy for glioma.

## The Oncogenic Roles of Endogenous PD-L1 as a Signaling Protein

**Figure 2:**
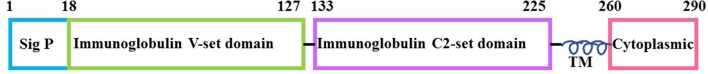
The structure of PD-L1 protein molecule. The numbers represent the amino acid. Sig P, signal peptide; it directs PD-L1 toward cell membrane; TM, transmembrane domain; Cytoplasmic: the cytoplasmic or intracellular domain of PD-L1. The intracellular domain contains several conserved motifs: RMLDVEKC, DTSSK and QFEET.

PD-L1 contains an extracellular immunoglobulin domain, a transmembrane domain (TM), and a short intracellular/cytoplasmic domain (30 aa), indicating its probability in controlling cell-intrinsic signaling (Figure 2). Earliest evidence of its intrinsic signaling activities was reported (Azuma et al., 2008). PD-L1 confers tumor resistance to CTL lysis, depending on its intracellular domain (Azuma et al., 2008). Similarly, Gato-Cañas et al. (2017) discovered that PD-L1 cell-intrinsic signaling protects cancer cells from IFN-induced cytotoxicity and accelerates tumor progression. They further identified three conserved motifs (i.e., RMLDVEKC, DTSSK and QFEET) in the intracellular domain of PD-L1 after aligning it in 10 mammalian species, revealing the functions of these motifs. By inhibiting signal transducers and activators of transcription 3 (STAT3) phosphorylation, RMLDVEKC motif of PD-L1 was required for cancer cells to withstand the IFN-induced apoptosis, while DTSSK motif counteracts the RMLDVEKC motif's function (Gato-Cañas et al., 2017). The evidence together demonstrates that PD-L1 confers resistance toward pro-apoptotic stimuli, such as IFN and CTL lysis, depending on its intracellular domain.

More direct evidence was provided by Chang et al. (2015). Tumor cells and T cells compete for nutrient such as glucose in tumor microenvironment, and predominant glucose consumption by tumors metabolically restricts T cell functions (e.g., reduction of mTOR activity/glycolytic capacity/IFN-γ production). *In vitro* and *in vivo* studies demonstrated that checkpoint blockade antibodies against PD-L1 restore glucose in tumor microenvironment, permitting T cell glycolysis and IFN-γ production. Further, *in vitro* studies showed that both PD-L1 antibodies and PD-L1 knockdown in tumor cells impaired sarcoma cell glycolysis and inhibited intracellular Akt/mTOR signaling, indicating the role of PD-L1 in modulating intracellular signaling pathway. Moreover, PD-L1 antibody treatment induces membranous PD-L1 internalization, implying that it is membranous PD-L1 rather than cytoplasmic PD-L1 that is involved in Akt/mTOR signaling in tumor cells (Chang et al., 2015). PD-L1 knockdown significantly decreases tumor volume of ovarian cancer and melanoma in immune-competent mice (Clark et al., 2016). PD-L1 knockout in murine medulloblastoma cells significantly reduced tumor incidence (30% of the mice inoculated with medulloblastoma cells remained tumor-free for more than 4 weeks) (Dorand et al., 2016). Recently, several teams reported that independent of immune function, PD-L1 regulated cell growth, proliferation, apoptosis, autophagy, migration and invasion in various cancers including ovarian cancer, melanoma, and pancreatic cancer via modulating PI3K/Akt/mTOR signaling pathway (Clark et al., 2016; Zhao et al., 2017). Additionally, PD-L1 promotes self-renewal and tumorigenicity of malignant melanoma initiating cells (Zheng et al., 2017), as well as embryonic stem cell transcriptional factors octamer-binding transcription factor 4 (OCT4) and Nanog expression dependent on PI3K/Akt activation and B lymphoma Mo-MLV insertion region 1 homolog (BMI1) expression in PI3K/Akt independent manner in breast cancer stem cells (Almozyan et al., 2017). Both evidence suggest the tumor-intrinsic role of PD-L1 in modulating signal transduction and the PD-L1's oncogenic/ tumorigenic role in certain cancers.

**Figure 3:**
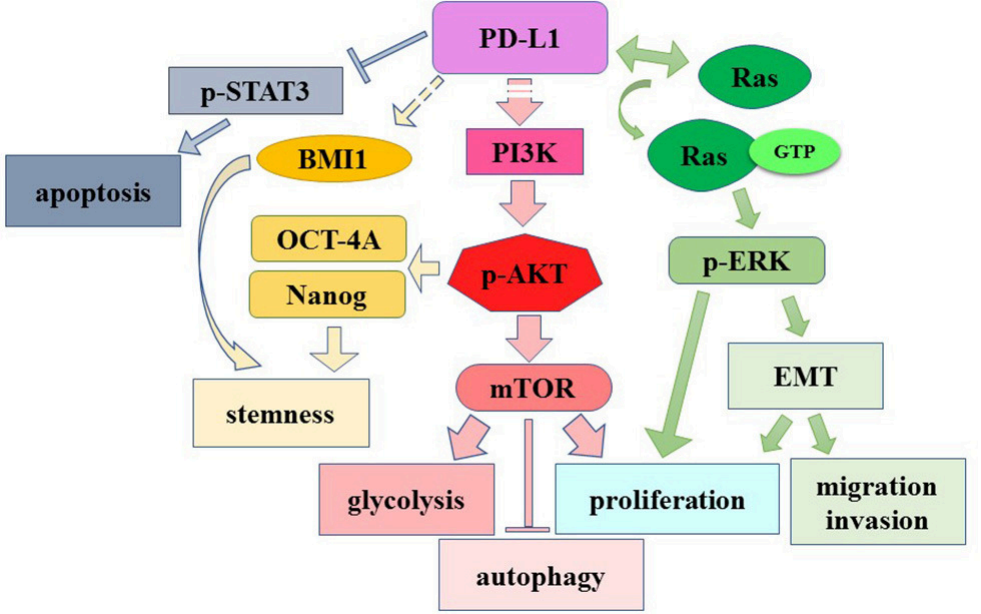
The role of cell-intrinsic PD-L1 in modulating signaling pathway. Dashed lines: unknown mechanisms. →, induction or activation; →, binding.

Specifically, in gliomas, PD-L1 expression was reported to be significant positively correlated to vascular endothelial growth factor (VEGF) and Ki-67 levels, detected by IHC with 64 patient specimens (Xue et al., 2017a). Our recent research reported that PD-L1 overexpression promoted and PD-L1 knockout/knockdown dampened glioma growth both *in vitro* and *in vivo* (Qiu et al., 2018). Further, transcriptome sequencing demonstrated that PD-L1 overexpression in glioma cell line significantly altered gene expression panel, suggesting PD-L1's cell-intrinsic roles. We have identified the intracellular interactions between PD-L1 and H-Ras, which further led to Ras/Erk activation, resulting in promoted glioma cell epithelial mesenchymal transition (EMT), migration and invasion. It is worth mentioning that PD-L1 knockdown almost abrogated the glioma xenografts formation in nude mice. This result suggests that the reduction of endogenous PD-L1 may be more effective than the employment of PD-L1 antibody, as the former inhibits both PD-1/PD-L1 axis signaling and oncogenic effects of PD-L1 (Qiu et al., 2018). It may also suggest an important glioma therapeutic strategy by drugs. Indeed, in glioma cells from the C57/GL261 orthotopic glioma model, we found that repaglinide significantly reduced PD-L1 expression, which correlates with the increase of TIL and the increase of survival time of mice (Xiao et al., 2017). These evidence suggest that targeting glioma PD-L1 directly by drugs or RNA interference might be an effective therapy for glioma. The functional roles of intrinsic PD-L1 as an oncogenic signaling molecule are summarized in Figure 3.

## The Effects of PD-1/PD-L1 Targeting-therapy in Glioma

Most clinical studies of PD-1/PD-L1 inhibition remain recruiting and inconclusive (Filley et al., 2017; Maxwell et al., 2017; Shergalis et al., 2018). Several studies reported that glioma or GBM patients may benefit from PD-1 antibody therapy. Reiss et al. reported that 24 HGG patients receiving pembrolizumab had a median PFS of 1.4 months and a median OS of 4 months with few serious toxicities (Reiss et al., 2017). Omuro et al. reported that nivolumab treatment on 3 in all 30 patients achieved partial responses, and 8 in all 40 patients with nivolumab treatment stayed stable for over 12 weeks (Omuro et al., 2018). Recently, Kline et al. reported that nivolumab in combination with reirradiation (*n* = 8) slightly prolonged OS of recurrent diffuse intrinsic pontine glioma in children compared to reirradiation alone (*n* = 4) (22.9 vs. 20.4 months since diagnosis; 6.8 vs. 6.0 months since reirradiation) in a retrospective study (Kline et al., 2018). All patients receiving reirradiation with or without nivolumab tolerated the therapy without severe acute or late toxicity. In a retrospective study of recurrent HGG among adult patients (*n* = 31), Kurz et al. reported that the median PFS showed no difference between patients receiving nivolumab (3.8 months) and pembrolizumab (2.3 months). The median survival time from date of first anti-PD-1 dose was 6.6 months (*n* = 31) (Kurz et al., 2018). In another retrospective study of recurrent HGG (*n* = 50), Mantica et al. reported a median PFS of 4.3 months and a median OS of 6.5 months after receiving nivolumab alone or in combination with bevacizumab (*n* = 43). In a subset of patients, disease stabilization appeared in heavily pre-treated recurrent HGG (Mantica et al., 2018).

There are case reports showing that PD-1 antibody therapy have evident therapeutic effects on GBM patients. Patrick et al. reported a 60-year-old GBM patient receiving nivolumab treatment for almost 2 years after standard radiotherapy and temozolomide therapy, and magnetic resonance imaging (MRI) revealed a continuous shrinking of the tumor (Roth et al., 2017). Bouffet et al. also reported that 2 young recurrent GBM patients receiving nivolumab treatment achieved 5 and 9 month-relieve with a MRI-detected shrinkage of GBM lesions (Bouffet et al., 2016). In addition, pembrolizumab treatment increases lymphocyte infiltration in resected metastatic spinal lesion, indicating that checkpoint PD1/PD-L1 blockade may result in immune activation in central nervous system (Johanns et al., 2016). In another case of an adult GBM patient, a severe hepatitis was found to be associated with nivolumab therapy, which may impair its therapeutic effect (Simonelli et al., 2016). The therapeutic effects of PD-1 antibody in glioma are summarized in Table 3.

**Table 3:** The therapeutic effects of PD-1 antibody in glioma patients.

**Glioma type**	**Antibody**	**Time to progression**		**Survival**		**References**
		**Months**	***P*-value**	**Months**	***P*-value**	
HGG	Pembrolizumab (*n* = 24)	1.4	range 0.2–9.4	4	range 0.5–13.8	Reiss et al., 2017
DIPG	reRT with Nivolumab vs. reRT (*n* = 31)	4.2 vs. 4.1	0.90	22.9 vs. 20.4	0.44	Kline et al., 2018
HGG	Nivolumab with Bevacizumab (*n* = 50)	4.3	95% CI (3.5–5.3)	6.5	95% CI (6.0–8.8)	Mantica et al., 2018
GBM	Nivolumab (*n* = 11) vs. Pembrolizumab (*n* = 19)	3.8 vs. 2.3	0.08	10.9 vs. 5.3	0.2	Kurz et al., 2018
GBM	Nivo3 (*n* = 10) vs. Nivo1+Ipi3 (*n* = 10) vs. Nivo3+Ipi1 (*n* = 20)	1.9 vs. 1.5 vs. 2.1	95% CI (1.3–4.6):(0.5–2.8): (1.4–2.8)	10.4 vs. 9.2 vs. 7.3	95% CI (4.1–22.8: 3.9–12.7: 4.7:12.9)	Omuro et al., 2018
GBM	Pembrolizumab (*n* = 10)			2.6 Media OS from start of PBZ	range 0.4–11.6	Blumenthal et al., 2016
GBM (bMMRD)	Nivolumab (*n* = 2)			>9		Bouffet et al., 2016
GBM	Nivolumab (*n* = 1)			>24		Roth et al., 2017

*reRT, reirradiation; Nivo3, nivolumab 3 mg/kg every 2 weeks; Nivo1+Ipi3, nivolumab 1 mg/kg + ipilimumab 3 mg/kg every 3 weeks for 4 doses, then nivolumab 3 mg/kg every 2 weeks; Nivo3+Ipi1, nivolumab 3 mg/kg + ipilimumab 1 mg/kg every 3 weeks for 4 doses, then nivolumab 3 mg/kg Q2W every 2 weeks*.

Until now, there has been no report of PD-L1 antibody therapy in glioma, however, animal studies support that PD-L1 antibody may also be effective in treatments of gliomas. Wainwright et al. reported that administration of PD-L1 antibody (clone 10F.9G2) had a long-term survival effect on mice/GL261 glioma model (60%, *n* = 10) (Wainwright et al., 2014). This effect was completely abolished in Rag1^−/−^ mice (lack functional T and B cells), implying that the therapeutic effect of PD-L1 antibody depends on immune response of glioma. However, PD-L1 antibody is unable to enhance animal survival time in a more aggressive B16-F10-derived intracranial melanoma, indicating a selective function of PD-L1 antibody in brain tumors. Reardon et al. also reported that PD-L1 antibody (339.6A2) had a long-term survival effect (25%, *n* = 8) in mice/GL261 glioma model, although slightly less potent compared to that of PD-1 antibody (Reardon et al., 2016). What is more, Saha et al. reported that administration of PD-L1 antibody (rat clone 10F.9G2) alone showed better therapeutic effects than PD-1 antibody alone, and it significantly increased mean survival time (42 days, *n* = 7; 25% increase) as compared to its mock (33.5 days, *n* = 6) in a glioma stem cell-derived glioma model (Saha et al., 2017).

PD-L1 antibody had cumulative therapeutic effects on C57 mice/GL261 orthotopic glioma when in combination with other drugs or CTLA4 antibody. Earlier supplement of methyltryptophan but not temozolomide together with PD-L1 antibody greatly enhances the long-term survival of mice bearing GL261-orthtopic glioma (Wainwright et al., 2014). Zhai et al. reported that PD-L1 in combination with CTLA4 antibodies significantly increased survival time of mice/GL261 intracranial glioma model (Zhai et al., 2017). Jiang et al. reported that intratumoral injection of oncolytic adenovirus (Delta-24-RGD expressing the immune costimulator OX40 ligand) followed by PD-L1 antibody (Bio X Cell) injection greatly improved the long-term survival rate of C57/Gl261 model mice (85%), compared to viruses (28%) or antibodies (15%) injection alone (Jiang et al., 2017). Moreover, most of the survived mice (*n* = 5/6) were long-term survived after re-implantation Gl261 cells in their contralateral hemispheres (rechallenge), indicating that the combination treatment induced developments of an immune memory which prevented tumor growth at a distant site. The actual therapeutic effect of PD-L1 antibody in glioma patients remains to be verified by clinical studies.

In summary, present data of clinical studies suggest that the overall effect of PD-1 antibody therapy in glioma is limited. However, its efficacy is much improved in some subtypes of glioma. The combination of immune therapy including PD-1/PD-L1 antibodies and molecular targeted anti-tumor drugs may be a future direction of glioma therapy.

## Conclusion

The positive rate and subcellular distribution of PD-L1 in glioma cells depend on the antibody that is used. The selection of a proper PD-L1 antibody is important in order to obtain positive results. PD-L1 is mainly upregulated in HGG and negatively correlated with the survival time of patients. Further analysis of the relationships between PD-L1 expression and other glioma molecular markers is worthy. Both positive (e.g., IFN-γ, IL-10) and negative (e.g., miR-34a, PTEN) signals are involved in the regulation of PD-L1 expression in gliomas. Comprehensive understanding of those regulatory mechanisms is beneficial for the modification of immunotherapy strategy. PD-L1 is not only associated with decreased CTL and increased Treg in glioma lesions, but also has intrinsic oncogenic roles by interacting with Ras. Intrinsic PD-L1 as well as its related signaling pathways may be also served as therapeutic targets. PD-1 antibody has limited therapeutic effects on glioma patients, while PD-L1 experimentally shows therapeutic effects in animal glioma-models. The improvement of PD-1/PD-L1 antibody-based immunotherapy relies largely on increasing clinical data. The combination of PD-1/PD-L1 antibodies with other molecule-targeted anti-cancer drugs is prospective.

## Author Contributions

RC: researched literature, wrote the manuscript and contributed to discussion; FL: researched literature and wrote the manuscript; XQ: researched literature, wrote the manuscript and modified the manuscript; XC: designed, wrote, reviewed, edited the manuscript, and contributed to discussion.

### Conflict of Interest Statement

The authors declare that the research was conducted in the absence of any commercial or financial relationships that could be construed as a potential conflict of interest.
